# The Treatment of Iritis

**Published:** 1899-07-15

**Authors:** 


### THE TREATMENT OF IRITIS.

Most cases of iritis which do not arise from injury or from an extension from some local inflammation, are but the expression of some dyscrasia, and the same may be said of the cyclitis which so generally accompanies inflammation of the iris. Yet, although general conditions, such as syphilis, gout, rheumatism, gonorrhœa, diabetes, tuberculosis, and a considerable variety of febrile diseases, may be the causes on which the attack of iritis primarily depends, there are certain general rules in regard to local treatment the observance of which are more important than any treatment directly aimed at the constitutional condition, even when this can be correctly ascertained. Mr. John Griffiths,[1] writing on the subject, says that no hard and fast lines can be laid down for the treatment of any given case of iritis, with this exception, that some mydriatic should be employed. This is essential, and outweighs the consideration of all constitutional treatment. In fact, he says it would be far better to adopt no other treatment beyond this local remedy than to treat the cause ever so well without using atropine. As a first essential, then, mydriasis is to be produced by a 1 per cent. solution of sulphate of atropine. Not only does this prevent adhesions of the iris to the capsule of the lens, a matter of paramount importance, but by paralysing the ciliary muscle as well as the sphincter pupillæ it secures complete intra-ocular rest, a suspension of all muscular movement within the eye, which is the first and highest aim in the treatment of acute inflammation. Atropine is then, without doubt, the drug *par excellence* for irido-cyclitis. Cocaine may perhaps assist its sedative action to some extent. Then the eye should be protected from the light, either by means of a broad shade, by the wearing of tinted goggles, or by the patient being kept in a darkened room. In the worst cases, in which the inflammation is the most acute and the conjuctiva chemosed, absolute rest in bed is most essential. Local depletion by the application of leeches to the temple gives almost immediate and usually permanent relief, and hot applications to the eye are also very serviceable. One point must be remembered about atropine, and that is the occasional existence of an idiosyncrasy in the patient in regard to its action. Atropine will in some cases produce severe local irritation, an inflammation of the skin and cellular tissue of the eyelids and cheek, accompanied often with a papular rash. Atropine may also set up a condition of poisoning, dryness of tongue, thirst, sleeplessness, &c. The latter can generally be prevented by keeping the lachrymal sac compressed for a few minutes after the drop has been instilled, but atropine irritation is more difficult to deal with, and, unfortunately, other mydriatics are apt to act in the same way. The hydrobromate of scopolamine appears to be the drug least likely to be attended with such a consequence. Passing now to those general measures which may be adopted almost irrespective of the constitutional cause which may happen to be at work in the individual case, it may be said that at the commencement of every attack of iritis a free purge should be administered; calomel and colocynth being given over-night, and a black draught in the morning. Alcohol should not be taken in any form, nor should smoking be allowed. Rest in bed must be ordered or not according to the severity of the case. In the more acute forms it is necessary, while in chronic forms of serous irido-cyclitis it would be wrong to keep the patient a prisoner. He must have outdoor exercise, and it may be necessary to send him to the seaside for a few weeks. Then, even where it may not be possible to satisfy oneself as to the constitutional cause, much may be done by adopting a treatment suitable to the condition of the patient. For the weak anæmic individual, a nourishing diet and a tonic regimen must be enforced, whereas the stout and plethoric must be reduced, and in all cases of doubt, if the general aspect of the patient does not forbid it, mercury should be given. "It is one of the grandest remedies in the treatment of most cases of irido-cyclitis, whether plastic or serous." As to constitutional treatment, in gouty cases diaphoresis must not be forgotten. This may be obtained either by giving pilocarpin hypodermically, or by a Turkish bath two or three times a week. In rheumatic cases salicylate of sodium or iodide of potassium may be given; in syphilis, of course, mercury; and in diabetes, in addition to local remedies, codeia should be given internally, along with a non-carbohydrate diet. In cases of tuberculous irido-cyclitis the question of excision must be carefully considered, and where this disease is found to be independent of any other tuberculous affection, excision should certainly be adopted as soon as the diagnosis is clear.

[1] Treatment, June 22.

